# Supplementary material for: Lung adenocarcinoma cells respond differently to mechanical stress in 3D versus 2D environments
Source: Commun Biol. 2025 Dec 11;8:1819. doi: 10.1038/s42003-025-09179-1 (PMC12749616; doi:10.1038/s42003-025-09179-1)
Supplement: Supplementary file 2 — Supplementary Information [file 42003_2025_9179_MOESM2_ESM.pdf]

Supplementary Information

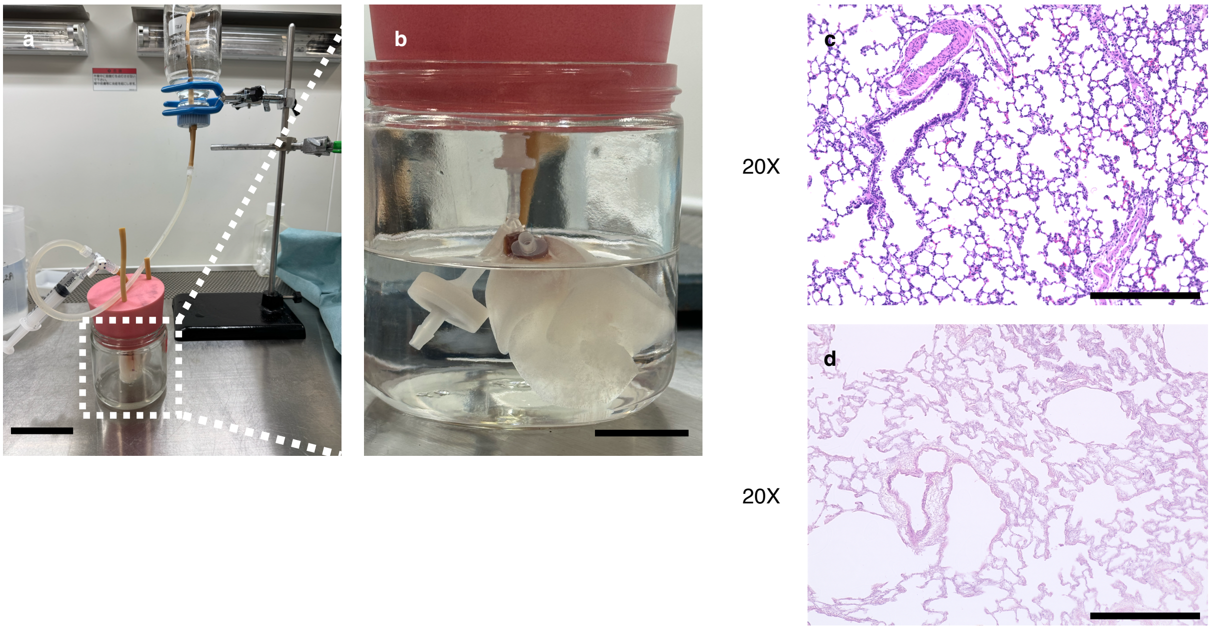

Supplementary Figure 1. Decellularisation system and histological findings. a, b) The lungs were placed in a beaker for decellularisation, and various solutions were gravity-fed through the tissues. **c**) HE staining of a naïve lung (objective lens: ×20). **d**) HE staining of a lung following decellularisation. All rat-derived cells were removed histologically, leaving only the structural framework that supports the alveoli (objective lens: ×20). Scale bars: (a) 6 cm; (b) 2 cm; (c, d) 200 μm. HE, haematoxylin and eosin.

Supplementary Movie 1. Airway reservoir liquid level response to RM. Timelapse recording of the airway reservoir under RM. Expansion and contraction of the lung in response to intrachamber pressure changes cause the movement of approximately 10 mL of culture medium in and out of the reservoir. RM, respiratory motion.

Supplementary Table 1. Comparison of RNA concentrations between decellularised lungs and sample lungs

Sample	RNA (ng/µl)	A260/280	A260/230
Decellularised lung	1.5	2.7	0.1
RM^{+} 1	116.7	2.0	2.1
RM^{+} 2	602.3	2.1	2.3
RM^{+} 3	327.9	2.1	2.2
RM^{+} 4	170.6	2.1	1.4
RM^{-} 1	111.1	2.0	2.2
RM^{-} 2	317.0	2.1	2.3
RM^{-} 3	618.2	2.1	2.2
RM^{-} 4	210.4	2.0	1.2

RM, Respiratory motion; RM^{+}, with RM; RM^{-}, without RM

Supplementary Table 2. Primer sequences used for qRT–PCR analysis

Species	Gene		Primer sequence (5'→3')
H. Sapiens	integrinβ1	Forward	TTGTGAAGCCAGCAACGGACAG
		Reverse	GCTCAGCACAGACACCAAGGC
H. Sapiens	CDKN1A	Forward	GAATTTGCCGTTGGGTCAAG
		Reverse	AGGAGAACACGGGATGAGGAG
H. Sapiens	NR4A3	Forward	ATAGTCTGAAAGGGAGGAGAGGTC
		Reverse	TCTGGGTGTTGAGTCTGTTAAAGC
H. Sapiens	CTGF	Forward	ACCGACTGGAAGACACGTTTG
		Reverse	CCAGGTCAGCTTCGCAAGG
H. Sapiens	EFNA1	Forward	GAAGGTGACTGTCAGTGGCAAA
		Reverse	TTGTCATGGGCCTGAGGACT
H. Sapiens	CXCL12	Forward	ATTCTCAACACTCCAAACTGTGC
		Reverse	ACTTTAGCTTCGGGTCAATGC
H. Sapiens	CCND1	Forward	CTGCCAGGAGCAGATCGAAG
		Reverse	AATGCTCCGGAGAGGAGGGAACT
H. Sapiens	LOX	Forward	TTCTTACCCAGCCGACCAAGATA
		Reverse	GTGTTGGCATCAAGCAGGTCA
H. Sapiens	GAPDH	Forward	CTCCTCCTGTTCGACAGTCAGC
		Reverse	CCCAATACGACCAAATCCGTT

qRT–PCR, quantitative reverse transcription polymerase chain reaction
