# Supplementary material for: Lung adenocarcinoma cells respond differently to mechanical stress in 3D versus 2D environments
Source: Commun Biol. 2025 Dec 11;8:1819. doi: 10.1038/s42003-025-09179-1 (PMC12749616; doi:10.1038/s42003-025-09179-1)
Supplement: Supplementary file 3 — Description of Additional Supplementary Files [file 42003_2025_9179_MOESM3_ESM.pdf]

Description of Additional Supplementary Files

File name: Supplementary Movie 1
Description: The timelapse shows culture medium moving in and out of the lung due to respiratory motion, causing visible changes in the liquid level of the airway reservoir
