# Supplementary material for: Lung adenocarcinoma cells respond differently to mechanical stress in 3D versus 2D environments
Source: Commun Biol. 2025 Dec 11;8:1819. doi: 10.1038/s42003-025-09179-1 (PMC12749616; doi:10.1038/s42003-025-09179-1)
Supplement: Supplementary file 5 — Reporting summary [file 42003_2025_9179_MOESM5_ESM.pdf]

Corresponding author(s): Naoya Kitamura, Tomoshi Tsuchiya

Last updated by author(s): Oct 19, 2025

Reporting Summary

Nature Portfolio wishes to improve the reproducibility of the work that we publish. This form provides structure for consistency and transparency in reporting. For further information on Nature Portfolio policies, see our Editorial Policies and the Editorial Policy Checklist.

Statistics

For all statistical analyses, confirm that the following items are present in the figure legend, table legend, main text, or Methods section.

n/a	Confirmed	
☐	☒	The exact sample size (*n*) for each experimental group/condition, given as a discrete number and unit of measurement
☐	☒	A statement on whether measurements were taken from distinct samples or whether the same sample was measured repeatedly
☐	☒	The statistical test(s) used AND whether they are one- or two-sided<br>*Only common tests should be described solely by name; describe more complex techniques in the Methods section.*
☐	☒	A description of all covariates tested
☐	☒	A description of any assumptions or corrections, such as tests of normality and adjustment for multiple comparisons
☐	☒	A full description of the statistical parameters including central tendency (e.g. means) or other basic estimates (e.g. regression coefficient) AND variation (e.g. standard deviation) or associated estimates of uncertainty (e.g. confidence intervals)
☐	☒	For null hypothesis testing, the test statistic (e.g. F, t, r) with confidence intervals, effect sizes, degrees of freedom and P value noted<br>*Give P values as exact values whenever suitable.*
☒	☐	For Bayesian analysis, information on the choice of priors and Markov chain Monte Carlo settings
☒	☐	For hierarchical and complex designs, identification of the appropriate level for tests and full reporting of outcomes
☒	☐	Estimates of effect sizes (e.g. Cohen's *d*, Pearson's *r*), indicating how they were calculated

Our web collection on statistics for biologists contains articles on many of the points above.

Software and code

Policy information about availability of computer code

Data collection	Not applicable.
Data analysis	Not applicable.

For manuscripts utilizing custom algorithms or software that are central to the research but not yet described in published literature, software must be made available to editors and reviewers. We strongly encourage code deposition in a community repository (e.g. GitHub). See the Nature Portfolio guidelines for submitting code & software for further information.

Data

Policy information about availability of data

All manuscripts must include a data availability statement. This statement should provide the following information, where applicable:

- Accession codes, unique identifiers, or web links for publicly available datasets
- A description of any restrictions on data availability
- For clinical datasets or third party data, please ensure that the statement adheres to our policy

The RNA-seq data generated in this study have been deposited in the Gene Expression Omnibus (GEO) under accession numbers GSE309015 and GSE309016, which will be made publicly available upon publication. The processed data, including lists of differentially expressed genes, as well as additional supporting datasets, have been deposited in Figshare (https://figshare.com/s/b55283770b3d1a28af20) and will be made publicly available upon publication. All other data are available from the corresponding author upon reasonable request.

All other data supporting the findings of this study are available from the corresponding author upon reasonable request.

Research involving human participants, their data, or biological material

Policy information about studies with human participants or human data. See also policy information about sex, gender (identity/presentation), and sexual orientation and race, ethnicity and racism.

Reporting on sex and gender	Not applicable.
Reporting on race, ethnicity, or other socially relevant groupings	Not applicable.
Population characteristics	Not applicable.
Recruitment	Not applicable.
Ethics oversight	Not applicable.

Note that full information on the approval of the study protocol must also be provided in the manuscript.

Field-specific reporting

Please select the one below that is the best fit for your research. If you are not sure, read the appropriate sections before making your selection.

☒ Life sciences ☐ Behavioural & social sciences ☐ Ecological, evolutionary & environmental sciences

For a reference copy of the document with all sections, see nature.com/documents/nr-reporting-summary-flat.pdf

Life sciences study design

All studies must disclose on these points even when the disclosure is negative.

Sample size	Sample sizes were predetermined based on similar previously published studies and resource availability.<br>• 2D culture experiments: n = 3<br>• 3D culture experiments: n = 4<br>• RNA sequencing: 2D, n = 2; 3D, n = 4<br>These numbers were sufficient to detect statistically significant differences using appropriate tests.
Data exclusions	No data were excluded from the analysis. All samples and replicates were included in the final statistical evaluation.
Replication	Key experiments were replicated independently at least three times for 2D cultures and four times for 3D cultures. Consistent results were obtained across replicates.
Randomization	Randomization was not applicable, as all samples were treated under strictly controlled experimental conditions in parallel groups (RM^+ and RM^-). No subjective selection was involved.
Blinding	Blinding was not performed. All analyses, including image quantification and gene expression assessment, were carried out using objective software-based methods to minimize bias.

Reporting for specific materials, systems and methods

We require information from authors about some types of materials, experimental systems and methods used in many studies. Here, indicate whether each material, system or method listed is relevant to your study. If you are not sure if a list item applies to your research, read the appropriate section before selecting a response.

Materials & experimental systems

n/a	Involved in the study
☐	☒ Antibodies
☐	☒ Eukaryotic cell lines
☒	☐ Palaeontology and archaeology
☐	☒ Animals and other organisms
☒	☐ Clinical data
☒	☐ Dual use research of concern
☒	☐ Plants

Methods

n/a	Involved in the study
☒	☐ ChIP-seq
☒	☐ Flow cytometry
☒	☐ MRI-based neuroimaging

Antibodies

Antibodies used	The primary antibodies used in this study were as follows: anti-Ki-67 (418071; Nichirei Biosciences, Tokyo, Japan), cleaved caspase-3 (25128-1-AP; Proteintech, IL, USA), β-catenin (84805; Cell Signaling Technology, MA, USA), integrin β1 (ab30394; Abcam, Cambridge, United Kingdom), and E-cadherin (20874-1-AP; Proteintech, IL, USA), fibronectin (ab314679; Abcam, Cambridge, United Kingdom), and type I collagen (28368; Cell Signaling Technology, MA, USA). All primary antibodies were diluted in SignalStain Antibody Diluent (Cell Signaling Technology) and incubated overnight at 4°C. Alexa Fluor 488- or 555-conjugated secondary antibodies (Thermo Fisher Scientific, MA, USA) were applied at room temperature for 1 hour.
Validation	Validation was not performed in this study. Antibodies and reagents were used according to the manufacturers' specifications and previously published protocols.

Eukaryotic cell lines

Policy information about cell lines and Sex and Gender in Research

Cell line source(s)	The human lung adenocarcinoma cell line A549 was obtained from the Japanese Collection of Research Bioresources (JCRB Cell Bank, Osaka, Japan).
Authentication	The A549 cell line was obtained from the JCRB Cell Bank (Osaka, Japan), which performs authentication using STR profiling. No additional authentication was conducted by the authors.
Mycoplasma contamination	Cells were not tested for mycoplasma contamination after receipt from the JCRB Cell Bank. However, the cell line was used within a short culture period and obtained from a reputable cell bank that routinely screens for contamination.
Commonly misidentified lines (See ICLAC register)	The A549 cell line used in this study is not listed among commonly misidentified cell lines according to the International Cell Line Authentication Committee (ICLAC) database.

Animals and other research organisms

Policy information about studies involving animals; ARRIVE guidelines recommended for reporting animal research, and Sex and Gender in Research

Laboratory animals	Decellularized lungs were obtained from 6–10-week-old male Sprague-Dawley rats (Japan SLC, Inc., Hamamatsu, Japan) after euthanasia, in accordance with institutional ethical guidelines for animal tissue use.
Wild animals	No wild animals were used in this study.
Reporting on sex	Only male rats (Sprague-Dawley, 6–10 weeks old) were used for lung decellularization. The A549 human lung adenocarcinoma cell line does not have a defined sex-specific phenotype, and sex differences were not assessed in this study.
Field-collected samples	No field-collected samples were used in this study.
Ethics oversight	All procedures involving animals were conducted in accordance with the institutional ethical guidelines of the University of Toyama for the use of animal tissues.

Note that full information on the approval of the study protocol must also be provided in the manuscript.

Plants

Seed stocks	No seed stocks were used in this study.
Novel plant genotypes	No novel plant genotypes were used in this study.
Authentication	Not applicable.
